# Unraveling Aspects of *Bacillus amyloliquefaciens* Mediated Enhanced Production of Rice under Biotic Stress of *Rhizoctonia solani*

**DOI:** 10.3389/fpls.2016.00587

**Published:** 2016-05-06

**Authors:** Suchi Srivastava, Vidisha Bist, Sonal Srivastava, Poonam C. Singh, Prabodh K. Trivedi, Mehar H. Asif, Puneet S. Chauhan, Chandra S. Nautiyal

**Affiliations:** ^1^Division of Plant Microbe Interactions, Council of Scientific and Industrial Research (CSIR)-National Botanical Research InstituteLucknow, India; ^2^Gene Expression Lab, Council of Scientific and Industrial Research (CSIR)-National Botanical Research InstituteLucknow, India

**Keywords:** *Rhizoctonia solani*, *B. amyloliquefaciens*, biological control, sheath blight, plant growth promotion

## Abstract

*Rhizoctonia solani* is a necrotrophic fungi causing sheath blight in rice leading to substantial loss in yield. Excessive and persistent use of preventive chemicals raises human health and environment safety concerns. As an alternative, use of biocontrol agents is highly recommended. In the present study, an abiotic stress tolerant, plant growth promoting rhizobacteria *Bacillus amyloliquefaciens* (SN13) is demonstrated to act as a biocontrol agent and enhance immune response against *R. solani* in rice by modulating various physiological, metabolic, and molecular functions. A sustained tolerance by SN13 primed plant over a longer period of time, post *R. solani* infection may be attributed to several unconventional aspects of the plants’ physiological status. The prolonged stress tolerance observed in presence of SN13 is characterized by (a) involvement of bacterial mycolytic enzymes, (b) sustained maintenance of elicitors to keep the immune system induced involving non-metabolizable sugars such as turanose besides the known elicitors, (c) a delicate balance of ROS and ROS scavengers through production of proline, mannitol, and arabitol and rare sugars like fructopyranose, β-D-glucopyranose and myoinositol and expression of ferric reductases and hypoxia induced proteins, (d) production of metabolites like quinazoline and expression of terpene synthase, and (e) hormonal cross talk. As the novel aspect of biological control this study highlights the role of rare sugars, maintenance of hypoxic conditions, and sucrose and starch metabolism in *B. amyloliquefaciens* (SN13) mediated sustained biotic stress tolerance in rice.

## Introduction

Rice is globally the second most important cereal after wheat catering to the calorific and nutritional needs of more than 40% of the global population. Many biotic stresses hamper rice production, specifically fungal diseases and cause huge economic losses. Among different fungal diseases, rice sheath blight caused by *Rhizoctonia solani* is a major production constraint causing annual yield losses up to 25–40% (Lee and Rush, 1983). The disease manifests initially as water soaked lesions on sheath of lower leaves and moves up the plant infecting both sheaths and leaves by joining the lesions (Lee and Rush, 1983; Kumar et al., 2009; XiaoXing et al., 2013). Conventional methods of introducing resistance to disease involve selection breeding, molecular breeding (XiaoXing et al., 2013; Hossain et al., 2014; Vasudevan et al., 2014) and development of transgenics through mapping and expressing different genes (Datta et al., 2001; Kalpana et al., 2006; Yadav et al., 2015). While the conventional breeding techniques are constraint by requirement of long time, development of transgenics becomes a matter of acceptance and propagation in many countries (Gewin, 2003). Therefore, quick alternatives used for disease management focuses on extensive use of fungicides, which creates concern about environmental health, pathogen resistance, and escalating costs (Slaton et al., 2003). Other alternatives include use of various plant extracts, microbial based products, and nutritional amendments for controlling the disease (Kumar et al., 2009; Carvalhais et al., 2013).

In the context of increasing concern for food and environmental safety, use of biocontrol agents and plant growth promoting rhizobacteria (PGPR) for reducing agro-chemical inputs in agriculture is considered as potentially sustainable means to control the disease (Herman et al., 2008; Srivastava et al., 2012; Nautiyal et al., 2013; Chowdhury et al., 2015). Microorganisms capable of directly antagonizing fungal pathogens by competing for the niche and essential nutrients, or by producing fungitoxic compounds (biofungicides) and inducing systemic acquired resistance are promising environment friendly methods for crop-management (Herman et al., 2008; Carvalhais et al., 2013; Nautiyal et al., 2013; Tóth and Stacey, 2015). Molecular studies on pathogenesis and stress related genes in rice cultivars have generated volumes of data and knowledge suggesting various signaling pathways and their regulation to play key roles in the crosstalk between plant and biotic/abiotic stresses for plant protection (Fujita et al., 2006; Zheng et al., 2013; Sayari et al., 2014). A lot of molecular and chemical cross talk is known to occur between a plant and the interacting microbe (de Souza et al., 2016). However, there may be unconventional mechanisms working in latency that may have a holistic effect in maintaining plant health. Since mutualistic plant–microbe associations are known to impart physiological and molecular benefits, they may be the constant source of plant health stimulant (Carvalhais et al., 2013; Tóth and Stacey, 2015). This interaction/cross talk of plant with a pathogen and a PGPR are though overlapping to some extent becomes specific depending on the nature of the interacting microbe at later stages (Pauly et al., 2006; Tóth and Stacey, 2015). Yet, there also exist a condition of tripartite interaction when a pathogen attacks a bacteria (biocontrol or PGPR) treated plant. We hypothesize a delicate balance between the pathways followed by the plant in presence of a pathogen or a PGPR and some latent mechanisms to combat disease incidence and growth promotion. This largely unexplored multivariate interaction among Plant–PGPR–Pathogen is the subject of the present study. The study presents a detailed analysis of molecular and physiological mechanisms modulated by plant growth promoting strain *Bacillus amyloliquefaciens* to explore the unconventional mechanisms employed by the rice plant against sheath blight.

## Materials and Methods

### *In Vitro* Fungus–Bacteria Interaction Assays

Biocontrol efficacy of *B. amyloliquefaciens* SN13 (Nautiyal et al., 2013) under *in vitro* condition was determined by growing the pathogen *R. solani* and PGPR SN13 on Potato Dextrose Agar (PDA) plates using dual culture technique and in different combinations in Czapek dox broth medium. The treatments were SN13 added 1 day before, SN13 and *R. solani* inoculated simultaneously and SN13 was added 1 day after *R. solani* inoculation. Culture supernatant after 3, 5, 7, and 10 days of inoculation was used for determination of protease, cellulase, β-1,3-glucanase and chitinase activity.

Proteolytic assay was carried out using 100 μl supernatant using casein as substrate and measured in terms of liberated tyrosine at 660 nm (Frey-Klett et al., 2011). Cellulolytic activity was determined by adding 100 μl supernatant to 1 ml of 0.5% carboxymethyl cellulose (CMC) prepared in 0.5 M phosphate buffer (pH 7.0) followed by 1 h incubation at 37°C. β-glucanase and chitinase assays were carried out by the co-incubation of 0.5% laminarin (prepared in 0.5 M phosphate buffer pH 7.0) and colloidal chitin in 50 mM acetate buffer along with the culture supernatant in 1:1 ratio at 37°C for 1 h. The liberated oligosaccharides were measured using dinitrosalicylic acid (DNS) at 540 nm.

### Greenhouse Studies

Role of *B. amyloliquefaciens* NBRISN13 in suppressing sheath blight disease in rice was assessed under greenhouse conditions. Susceptible rice cultivar *Narayan* and the virulent isolate of sheath blight pathogen *R. solani* were used in all the experiments. Soil application of *B. amyloliquefaciens* NBRISN13 and transplantation of 15 days old seedlings was carried out as described earlier (Nautiyal et al., 2013). Plants were inoculated with the pathogen *R. solani* at maximum tillering stage (45 days after transplantation) by placing sclerotia wrapped in moist absorbent cotton at the lowest inner sheath of the main tiller and the plants were covered with inverted polythene bags to maintain humidity for 48 h to ensure pathogen infection (Marshall and Rush, 1980; Zheng and Wang, 2011). Treatments were (a) SN13 (b) *R. solani* and (c) SN13 + *R. solani* (d) control. Observations were recorded for (a) development and severity of sheath blight symptoms at 15, 30, and 45 days post inoculation (dpi) (b) microarray analysis and GCMS profiling at 45 dpi.

### Histological Assays

Plants harvested at 15 dpi were used for microscopic examinations. The leaf samples were fixed in 70% ethanol:acetic acid (3:1) for 12 h followed by two washings for 1 h each in 70% ethanol. The fixed samples were preserved in 70% ethanol and stored at 4°C until use. The outermost covering of the stem was stained with 0.1% trypan blue to study surface colonization of the pathogen. Application of stain was followed by heat treatment in a microwave at the high energy level (∼500 watts) for 10 s in flooded condition. The sample was washed and destained in 70% ethanol for 10 min and observed under microscope. To study the starch distribution in stem, hand cut transverse sections were stained with Lugol’s solution (0.01% KI) which turned the starch granules blue to black. Brown colored granules were indicative of inhibition of KI and starch reaction due to presence of organic acids or phenolic compounds such as ascorbic, gallic, and chlorogenic acid as suggested earlier (Kurata and Yamamoto, 1998).

### Sugar, Chlorophyll, and Proline Content in Greenhouse Grown Plants

Sugar estimation in greenhouse grown plants was performed using 0.2 g of fresh leaves crushed with 80% methanol as per the protocol of Dubois et al. (1956). Total chlorophyll and proline content were determined as described earlier by Nautiyal et al. (2013).

### Defense Enzyme Assays

Defense enzyme assays, i.e., superoxide dismutase (SOD), ascorbate peroxidase (APX), and catalase (CAT) were performed after crushing 1.0 g of plant tissue in extraction buffer containing 0.1 M potassium phosphate buffer, 0.1 mM EDTA, 1% polyvinylpyrrolidone (PVP), PMSF (protease inhibitor), and dithiothreitol.

Superoxide dismutase activity was determined by measuring the inhibition of photochemical reduction of nitroblue tetrazolium (NBT; Beauchamp and Fridovich, 1971). APX activity of the samples was quantified as per the method of Nakano and Asada (1981). The enzyme activity was expressed as μmoles of ascorbate oxidized (𝜀 = 2.8 mM^−1^ cm^−1^) per minute per gram fresh weight. Catalase activity was determined at 25°C according to Aebi (1984). The enzyme activity was expressed as μmoles of H_2_O_2_ degradation per minute per gram of fresh weight. All enzyme activities were converted into Units mg^−1^ protein.

### Cell Wall Degrading Enzyme Assays

Rice leaves were homogenized in sodium phosphate buffer (pH 7.0) containing 20 mM cysteine HCl, 20 mM EDTA and 0.5% triton X-100 and different cell wall degrading enzymes (CWDE) like polygalacturonase (PG), pectin methyl esterase (PME) and endopectate lyase (PL) and β-glucosidase (β-gluc) activity in the homogenate was measured as described earlier (Nautiyal et al., 2013).

### Hormone Analysis

Different hormones such as gibberellic acid (GA), indole-3-acetic acid (IAA), abscisic acid (ABA), and salicylic acid (SA) were analyzed by modified method of Wu and Hu (2009) using HPLC. Primary stock solutions (1 mg ml^−1^) of GA, IAA, ABA, and SA were prepared and diluted (0.5–50 μg ml^−1^) in methanol. All solutions were stored at −20°C until use.

Leaf tissues were homogenized with 70% methanol with constant overnight stirring at 4°C and evaporated on rotavapor under vacuum. The remaining aqueous phase was adjusted to pH 8.5 with phosphate buffer (pH 8.5) and partitioned with ethyl acetate thrice. Ethyl acetate phase was removed and aqueous phase was adjusted to pH 2.5 using HCl and partitioned with diethyl ether. Diethyl ether fraction was evaporated to dryness and dissolved in 2 ml of methanol and used for HPLC analysis.

Qualitative and quantitative analysis for separation of all compounds was achieved by HPLC-PDA with a LC-10 system comprising LC-10AT dual pump system, SPD-M20A PDA detector, and rheodyne injection valve furnished with a 20 μl sample loop (Shimadzu, Japan). Compounds were separated on a 250 mm × 4.6 mm, i.d., and 5 μm pore size Merck RP-C18 column protected by guard column containing the same packing. Mobile phase was isocratic, consisting of 30 mM orthophosphoric acid in HPLC-grade water (component A) and acetonitrile (component B) in 70:30 ratios. HPLC was run at the flow rate of 0.8 ml min^−1^ for 30 min. Data was recorded and analyzed on different wavelength viz. IAA at 265 with 12.50 retention time (RT), ABA, and GA at 208 with 14.43 and 6.31 RT and SA at 280 nm with a RT of 15.92. Data was integrated by Shimadzu class VP series software after comparing with standards, results were mean values of three replicates (**Supplementary Figure S2**).

### GC–MS Analysis

Gas chromatography–mass spectrometry (GC–MS) analysis of the rice leaves was performed using methanolic extract of rice leaves as described earlier (Fukusaki et al., 2006). Fresh rice leaves (1 g) were extracted in 5 ml of methanol and water mixture (2.5:1v/v). The polar phase was lyophilized to dryness and TMS derivatives were prepared. The derivatized mixture was analyzed on Gas Chromatography–Mass Spectrometry (GC–EIMS) on a Thermo Fisher TRACE GC ULTRA coupled with DSQ II Mass Spectrometer using TR 50MS column (30 m × 0.25 mm ID × 0.25 μm, film thickness). Conditions used were – carrier gas: Helium; flow rate: 1 ml min^−1^; injector temperature: 230°C; oven temperature: started from 70°C, (hold time 5.0 min) to 290°C with ramp of 5°C min^−1^ (hold time 5 min); sample injection: split mode (1:50); injection volume: 1 μl; ion source temperature: 220°C; transfer line temperature: 300°C and ionization: electron impact mode at an ionization voltage of −70 eV. Mass range was used from *m/z* 50 to 650 amu (**Supplementary Figure S3**). Identification of individual compounds was carried out by comparison of their mass spectra with those of the internal reference mass spectra library (NIST/Wiley).

### Microarray Analysis

Total RNA was isolated from liquid N_2_ frozen leaf blade tissues of each treatment using the RNeasy Plant Mini Kit (Qiagen, Hilden, Germany). Three independent replicated microarray analysis experiments were carried out using Affymetrix GeneChip^®^ Rice Genome Arrays (Gene Expression Omnibus platform Accession No. GPL2025). Preparation of targets, arrays hybridization, etc., were carried out according to manufacturer’s instructions (Affymetrix, USA) and analyzed using Affymetrix GeneChip Operating Software (GCOS version 1.3). Normalization and differential expression analysis was carried out using dChip software (DNA-chip Analyser). Significant differentially expressed genes were selected after a combined criterion of >twofold at *P* < 0.05 in the *t*-tests after normalization at different level.

### Real Time PCR Analysis

The same total RNA was used for first strand cDNA synthesis using maxima H minus first strand cDNA synthesis kit (Fermentas, Thermo scientific) as per manufacturer’s instructions. Real-time PCR for randomly selected genes using actin as an internal reference gene on an Agilent technology, Stratagene Mx3000P Quanti Tect^TM^ SYBR^®^ Green PCR kit (Qiagen) (Supplementary Table S1). The reactions were performed using the cycle conditions of an initial denaturation at 94°C for 5 min, followed by 35 cycles of 94°C for 30 s, 60°C for 30 s, and 72°C for 30 s. After obtaining *c*t-value for each reaction, the fold change was calculated by using delta–delta *c*t method.

### Statistical Analysis

Plants were harvested 45 dpi. Values are means ± SE of 12 replicates from each treatment of rice. Different biochemical assays were performed at *n* = 3. Tukey’s multiple HSD test at *P* = 0.05 was used to evaluate significance of the data and indicated with different letters. Principal component analysis (PCA) for metabolic differentiation among the treatments was analyzed using Statistica 7.0. All the comparisons were made as compared to control.

## Results

### Biocontrol Efficacy of *B. amyloliquefaciens* under *In Vitro* Conditions

**FIGURE 1:**
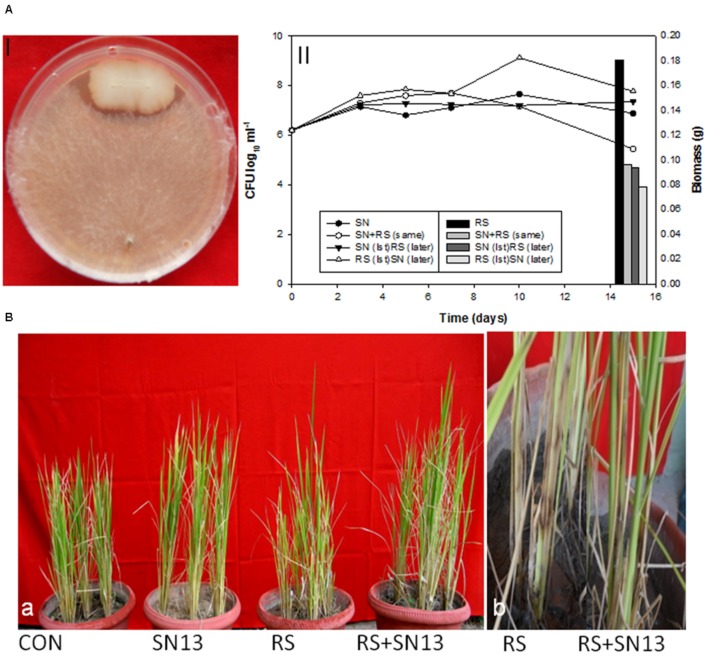
**(A)** Biocontrol efficacy of *Bacillus amyloliquefaciens* (SN13) against *Rhizoctonia solani* (*R. solani*) in dual culture method (I) and broth conditions (II) (primary axis) SN13 growth, (secondary axis) *R. solani* biomass. **(B)** Effect of SN13 on plant growth and biocontrol of rice sheath blight (a) 45 dpi (b) close view of lesion development in RS and SN13 + RS at 15 dpi. CON, control; SN13, biocontrol agent; *R. solani*, pathogen; dpi, days post infection.

**FIGURE 2:**
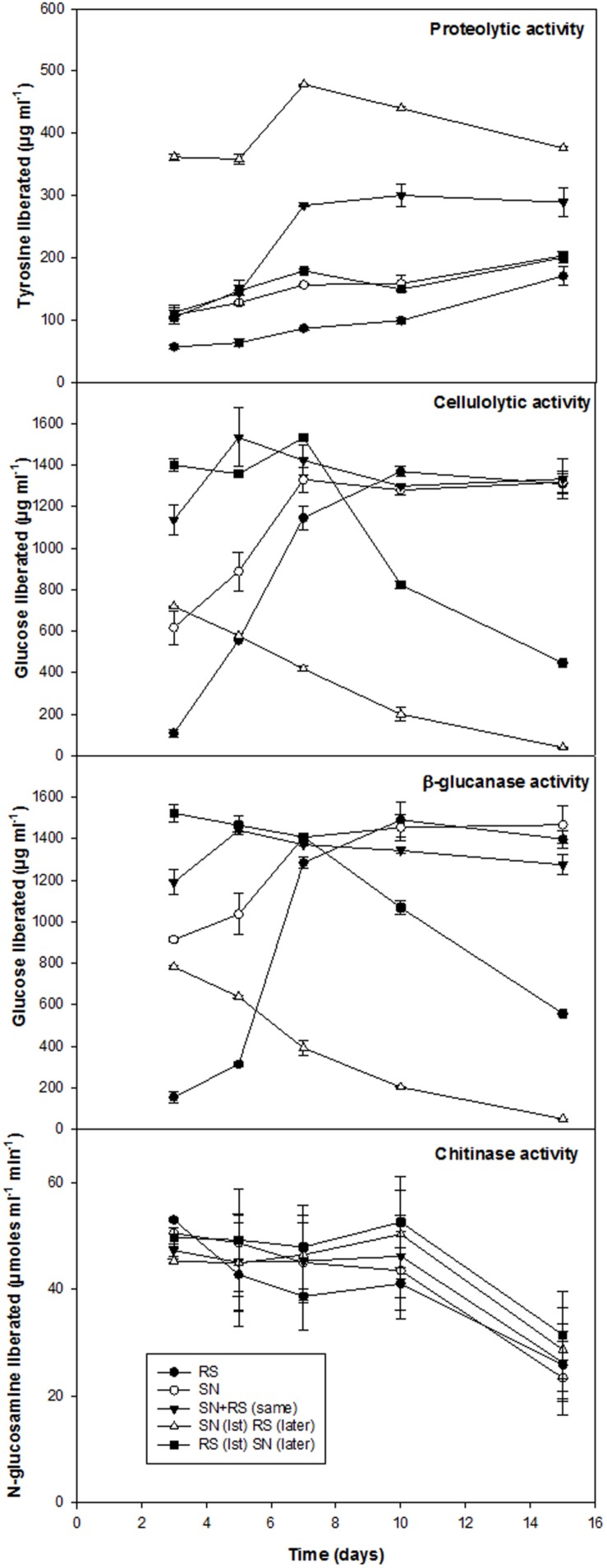
**Enzymatic profile of medium supernatant during *in vitro* interactions of SN13 and *R. solani***.

Biocontrol efficacy of *B. amyloliquefaciens* (SN13) against necrotrophic pathogen *R. solani* initially evaluated as dual culture method was further validated by co-incubating the *R. solani* and SN13 under *in vitro* conditions. Interaction studies showed that co-incubation results in ∼50% decrease in fungal dry mass irrespective of the combination strategies used (i) SN13 added 1 day before *R. solani* inoculation (ii) SN13 and *R. solani* inoculated simultaneously and (iii) SN13 added 1 day after *R. solani* inoculation (**Figure 1A**). SN13 showed profuse growth in all the three combinations (**Figure 1A**). Mycolytic enzymes assessed as a known mechanism of biocontrol showed synergistically enhanced protease (∼70–120%) and chitinase (∼2–21%) activity when SN13 and *R. solani* were co-inoculated as compared to *R. solani* or SN13 alone. Among the three combinations pre-inoculation of SN13 in SN13 + *R. solani* combination was found to enhance protease and chitinase activity by 120 and 21%, respectively (**Figure 2**). Present study also reports reduction in glucanase and cellulase activity (2–96%) in *R. solani* + SN13 combinations. Higher protease and chitinase activity probably proves the antifungal activity of the strain NBRISN13 associated with their secretory systems for induced immune response.

### Biocontrol Efficacy of *B. amyloliquefaciens* against *R. solani* in Rice

The disease incidence observations were taken in terms of number, diameter, and length of the spots and their distance from the girth. The lesions observed were of necrotrophic type and were increasingly high in the *R. solani* treatment (**Figure 1B**). The number and size of spots consistently increased till 45 days post infection (dpi) in the *R. solani* treatment (9–11 in number and 2.8–3.8 mm in size). Reduction in disease incidence in terms of number and size in SN13 + *R. solani* treatment manifested its efficacy as a biocontrol agent under natural conditions (**Table 1**).

**Table 1:** Effect of *B. amyloliquefaciens* SN13 treatment on plant growth promotion and disease incidence of *R. solani* on *O. sativa* plants.

Parameters/treatments	Days after fungal inoculation (dpi)	CON	*R. solani*	SN13	SN13 + *R. solani*	CD at 5%
**Disease index**						
No. of spots	15	nd	8.83 ± 0.29	0.00 ± 0.00	8.17 ± 0.56	1.09
	30	nd	9.83 ± 0.50	0.00 ± 0.00	6.08 ± 0.35	1.02
	45	nd	10.58 ± 0.76	0.00 ± 0.00	7.83 ± 0.40	0.55
Length of spots (cm)	15	nd	2.83 ± 0.31	0.00 ± 0.00	2.82 ± 0.44	0.69
	30	nd	3.24 ± 0.16	0.00 ± 0.00	2.04 ± 0.20	0.41
	45	nd	3.88 ± 0.46	0.00 ± 0.00	2.38 ± 0.16	0.30
Distance from the girth(cm)	15	nd	6.98 ± 0.89	0.00 ± 0.00	6.98 ± 0.06	1.41
	30	nd	24.79 ± 1.51	0.00 ± 0.00	22.23 ± 2.20	2.79
	45	nd	21.79 ± 0.96	0.00 ± 0.00	19.62 ± 1.39	1.78
Diameter of spots (cm)	15	nd	1.58 ± 0.18	0 ± 0.00	1.22 ± 0.15	0.25
	30	nd	0.49 ± 0.03	0 ± 0.00	0.23 ± 0.02	0.07
	45	nd	0.55 ± 0.03	0 ± 0.00	0.48 ± 0.03	0.05
**Biochemical characterization**						
Chl A (mg/g)	15	0.07 ± 0.00^a^	0.12 ± 0.00^c^	0.15 ± 0.00^d^	0.10 ± 0.00^b^	
	30	0.06 ± 0.00^a^	0.07 ± 0.00^b^	0.09 ± 0.00^d^	0.07 ± 0.00^c^	
	45	0.05 ± 0.00^a^	0.05 ± 0.00^b^	0.07 ± 0.00^d^	0.06 ± 0.00^c^	
ChlB (mg/g)	15	0.025 ± 0.00^a^	0.036 ± 0.00^bc^	0.04 ± 0.00^c^	0.032 ± 0.00^ab^	
	30	0.022 ± 0.00^a^	0.04 ± 0.00^b^	0.05 ± 0.00^c^	0.034 ± 0.00^b^	
	45	0.02 ± 0.00^a^	0.03 ± 0.001^b^	0.04 ± 0.00^d^	0.03 ± 0.00^c^	
Total Chl (mg/g)	15	0.09 ± 0.00^a^	0.16 ± 0.00^c^	0.19 ± 0.00^d^	0.13 ± 0.00^b^	
	30	0.08 ± 0.00^a^	0.10 ± 0.00^b^	0.14 ± 0.00^c^	0.11 ± 0.00^b^	
	45	0.07 ± 0.00^a^	0.08 ± 0.00^b^	0.12 ± 0.00^d^	0.12 ± 0.00^c^	
Sugar (μg/gm)	15	45.7 ± 0.98^a^	57.2 ± 3.92^b^	82.2 ± 2.77^c^	64.4 ± 4.95^b^	
	30	64.3 ± 1.32^a^	266.7 ± 5.23^b^	380.9 ± 1.67^c^	62.7 ± 0.40^a^	
	45	58.2 ± 1.6^a^	153.3 ± 0.9^b^	124.9 ± 0.9^c^	243.9 ± 1.3^a^	
Proline (μM)	15	10.34 ± 0.3^a^	10.83 ± 0.095^a^	16.39 ± 2.41^b^	29.20 ± 0.85^c^	
	30	5.11 ± 0.41^b^	11.27 ± 0.15^c^	3.13 ± 0.15^a^	27.61 ± 0.69^d^	
	45	3.46 ± 0.05^b^	5.94 ± 0.00^c^	7.48 ± 0.55^a^	3.25 ± 0.16^d^	
**Physical parameters**						
Root length (cm)		14.39 ± 0.74^b^	14.13 ± 1.32^b^	14.90 ± 1.06^b^	10.46 ± 0.90^a^	
Shoot length (cm)		59.02 ± 2.89^a^	52.96 ± 3.18^a^	67.13 ± 2.17^b^	68.92 ± 3.02^b^	
No of Spikes	45	1.08 ± 0.25^a^	1.00 ± 0.24^a^	1.58 ± 0.19^a^	1.17 ± 0.24^a^	
No of Tillers		1.08 ± 0.08^a^	1.08 ± 0.08^a^	1.33 ± 0.18^a^	1.16 ± 0.11^a^	
Dry weight (gm)		5.29 ± 0.37^b^	3.37 ± 0.54^a^	7.44 ± 0.25^d^	6.57 ± 0.77^c^	

*Different letters on bar diagram show significant differences at p < 0.05 using Tukey’s test. nd, not detected.*

The physical growth parameters of the rice plants showed 15.25% reduction in shoot length on *R. solani* infection which was increased in SN13 + *R. solani* treatment by 23.72% as compared to control. The reduction in dry weight (36.29%) observed in *R. solani* alone treatment was overcome by 24.19% in SN13 + *R. solani* treatment. However, SN13 alone treatment was found to increase the dry mass by 40.64% as compared to control (**Figure 1B**).

### Histological Studies

A profuse colonization of stem surface with hyphae penetrating the stem tissues was observed in *R. solani* treatment. Whereas, SN13 primed plants showed very sparse *R. solani* colonization, restricted stem surface in SN13 + *R. solani* treatment (**Supplementary Figure S1**).

**FIGURE 3:**
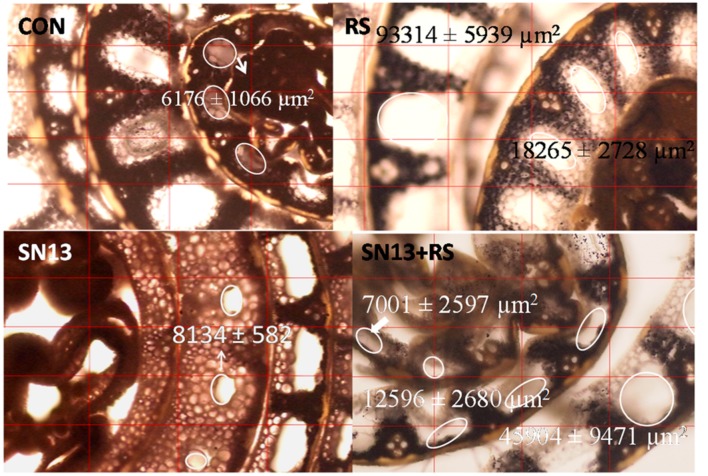
**Stem cross section showing effect of ***B. amyloliquefaciens*** (SN13) on aerenchyma formation in rice, 15 dpi.** “First’ and ‘second’ indicate the sheath layer from core and the encircled area shows the extent of aerenchyma formation. CON, control; SN13, biocontrol agent; *R. solani*, pathogen; dpi, days post infection. Magnification 40×.

Programmed cell death (PCD), an important phenomenon results in aerenchyma formation which is more progressive under stress conditions. Large sized aerenchyma are observed in outer sheath which are decreasing in size toward inner layers. The air spaces in aerenchyma, encircled in **Figure 3** were observed to increase in presence of *R. solani* with an average area ranging from 18000 and 94000 μm^2^ as compared to the control (6000–8500 μm^2^). SN13 treatment reduced/delayed formation of aerenchyma in the middle sheath which ranged from 0 and 8000 μm^2^ and was nearly absent in the innermost layer. The observations from stem sections stained with IKI showed loss of iodine reactivity with starch granules in SN13 treatment showing brown colored cells, whereas black colored starch granules were distinctly observed in *R. solani* and SN13 + *R. solani* treatments.

### Effect of *B. amyloliquefaciens* Inoculation on Physiological Parameters of Rice Plants

Physiological status of SN13, *R. solani*, and SN13 + *R. solani* treated plants was determined by measuring chlorophyll, sugar, and proline content. Significant changes in Chl A and total chlorophyll content was observed with time. Chlorophyll content decreased progressively in all the treatments, however, with different rates. The per cent reduction with time was maximum in *R. solani* treatment (50%) was followed by SN13 (36.8%) and SN13 + *R. solani* (7.6%). Higher sugar content in shoots of SN13 treated plants was observed over the period of 15–45 dpi. At the time of harvest the sugar content of SN13 + *R. solani* treated leaves was found to be highest by 320% and less in individual treatments. The pathogen and PGPR alone were found to increase proline content by 70 and 114%, respectively. However, SN13 + *R. solani* did not show change in proline content with respect to control (**Table 1**). All the comparisons were made as compared to control.

### Modulation in Defense Enzyme Activities

**FIGURE 4:**
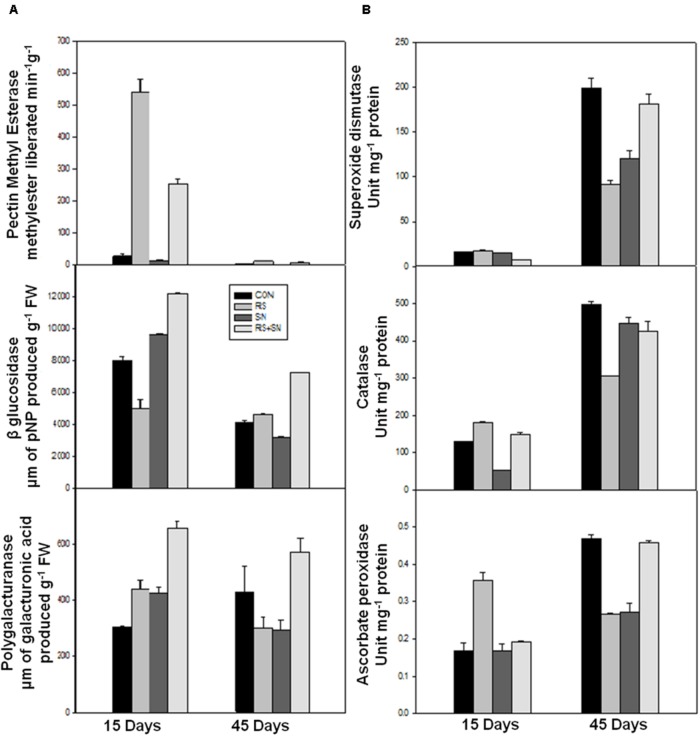
**Effect of ***B. amyloliquefaciens*** (SN13) inoculation on cell wall degrading enzyme (A) and defense enzyme (B) activities 15 and 45 dpi in rice leaves.** CON, control; SN13, biocontrol agent; *R. solani*, pathogen.

Defense enzymes were assayed in rice leaves after 15 and 45 dpi for CAT, APX, and SOD activity. These activities were found to be higher in *R. solani* treatment by 39, 11, and 12%, respectively. The activities were more pronounced at 15 dpi as compared to 45 dpi (**Figure 4B**). SN13 imparted unstressed condition to the plant which maintained slightly higher or unaltered defense enzyme activities in presence and absence of pathogen treatments (SN13, SN13 + *R. solani*) as compared to control.

### Modulation in Cell Wall Enzyme Activities

Polygalacturonases, PME, and β-glucosidases (β-gluc) activities were measured in rice plants at 15 and 45 dpi. There was 11 and 179% increase in the activities of PME and β-glucosidase, except PG (46% decrease) in the presence of *R. solani* at 45 dpi (**Figure 4A**). However, at 15 dpi, PME and PG activities were increased by 1800 and 45%, respectively, and accompanied by 37% reduction of β-glucosidase activity in *R. solani* treatment. SN13 + *R. solani* treatment at 45 dpi showed reduction in PME activity by 100% whereas PG and β-gluc activity were enhanced by 33 and 77%, respectively, but at 15 dpi all the three activities were higher in SN13 + *R. solani* treatment as compared to control (**Figure 4A**).

### Hormone Accumulation

**FIGURE 5:**
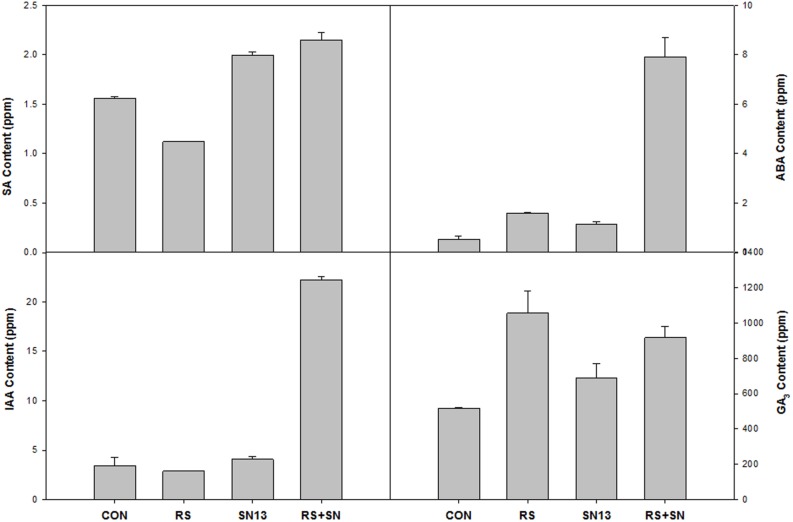
**Effect of ***B. amyloliquefaciens*** (SN13) inoculation on hormone synthesis at 15 dpi in rice leaves.** CON, control; SN13, biocontrol agent; *R. solani*, pathogen.

HPLC analysis of plant hormones IAA, GA3, SA, and ABA showed differential accumulation in the treatments emphasizing the involvement of hormonal cross talk during tripartite interaction of PGPR–pathogen and rice (**Figure 5**). Reduced accumulation of SA in *R. solani* treatment (21.3%) was found to be enhanced by the presence of SN13 (SN13 + *R. solani*) by 36.8%. IAA content was found to be higher by 450% in SN13 + *R. solani* treatment which was otherwise unaltered in other treatments. *R. solani* treatment was found to have GA3 and ABA nearly twice the amount as in control. ABA concentration, approximately 10 times higher than that of control was observed in SN13 + *R. solani* treatment.

### Effect of *B. amyloliquefaciens* and *R. solani* Treatment on Metabolic Profiling of Rice

Gas chromatography–mass spectrometry analysis depicted the metabolic modulations in rice leaves treated in response to the interactions with SN13 and *R. solani* (**Figure 8**; **Supplementary Figures S3** and S6 and Supplementary Table S2). The defense responsive phenolic acids, sugar, and their analogs and alkaloids were found to be differentially accumulated in *R. solani*, SN13 and combination treatments. *R. solani* infection led to higher accumulation of several defense responsive metabolites which were otherwise less accumulated in SN13 and SN13 + *R. solani* treatments. *R. solani* infection lead to enhanced or exclusive production of propionic acid, succinic acid, and quinoline which are known members of the phenylpropanoid pathway. ROS quenching metabolites pyrazole, imidazole, and chlorothiophene were triggered in *R. solani* treatment in response to defense. Besides, SAR inducers, quinoline, an alkaloid plant secondary metabolite was induced in response to *R. solani* infection (**Figure 8**; **Supplementary Figure S6**; Supplementary Table S2). Several sugars and sugar alcohols were observed in enhanced amounts during *R. solani* infection which included glycerol, arabitol and mannitol (**Figure 8**). Significant reductions as compared to control were observed with respect to concentration of turanose, mannopyranose, and sucrose during pathogen treatment, which were further enhanced in SN13 + *R. solani* treatment (Supplementary Table S2). Fructopyranose, glucopyranose, and myoinositol are the discriminating sugars accumulating higher in SN13 + *R. solani* treatment (**Figure 8**). Enhanced level of glucose/fructose ratio in SN13 + *R. solani* may be because of stimulated breakdown of sucrose into glucose and fructose. Some discriminating factors like quinazoline, a defense induced alkaloid were specifically accumulated in higher amounts in SN13 + *R. solani* treatment (**Figure 8**).

Principal component analysis analysis of 20 metabolites from rice shoots in four treatments (CON, SN13, *R. solani*, SN13 + *R. solani*) which distributed at 84.25 and 12.52% on factor 1 and 2, respectively, infer the distinct pattern among four treatments. Results clearly reveal that metabolic profiles of SN13 and SN13 + *R. solani* are closer to each other as compared to control and *R. solani* (**Supplementary Figure S6**). From these results it is interpreted that inoculation of SN13 altered the metabolic profiles in *R. solani* infected rice plants resulting in amelioration of the biotic stress.

Metabolites based PCA clearly show a wide distribution among some metabolites from the four treatments that are distributed at 61.41 and 25.46% on factor 1 and 2, respectively. Nine metabolites, i.e., propanoic acid (Pro), silanamine (Sil), 1H-imidazole (Imi), succinic acid (Suca), glycerol (Gly), arabitol (Ara), mannitol (Man), 2,5-di-butyl-3-chlorothiophene (Dbcn), quinoline (Qui) were grouped together on the plot, predicting that they remained unaltered in the four treatments. Other 11 metabolites, i.e., 1H-pyrazole (Pyr), fructopyranose (Fru), D-fructose (Dfr), D-glucose (Glu), mannopyranose (mnp), β-D-glucopyranose (Glp), myoinositol (Myo), sucrose (Suc), turanose (Tur), 1,2-benzene dicarboxylic acid (Ben), quinazoline (Qun) were distinctly separated on the plot (**Figure 8**).

### Effect of *B. amyloliquefaciens* and *R. solani* Treatment on Expression Profiling

**FIGURE 6:**
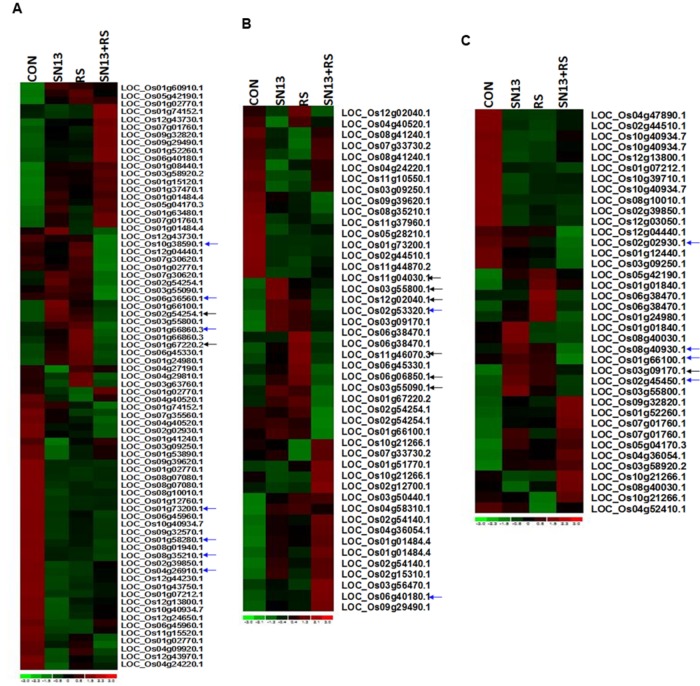
**Heat map showing expression profiling of differentially expressed genes of primary metabolism (A), stress response (B), and hormone biosynthetic pathway (C) in rice leaves.** CON, control; SN13, biocontrol agent; *R. solani*, pathogen. Black arrows indicate the marked genes restored as control as compared to *R. solani* infection. Blue arrows indicate some of the genes randomly selected for RT-PCR analysis.

Differentially expressed genes through microarray analysis were identified by dchip analysis. The comparisons were considered as control vs. SN13, control vs. *R. solani* and control vs. SN13 + *R. solani*. While comparing control vs. SN13, only 63 genes showed significant differential expression with *p*-value of >0.005. Of these, most of the genes were down-regulated and only 18 genes showed >onefold expression. Significantly expressed genes were: putative pullulanase precursor, α-amylase, 4-α-glucanotransferase, α-glucan phosphorylase isozyme, α-amylase, catalytic domain containing protein, universal stress protein domain containing protein, NADPH-dependent FMN reductase domain containing protein, inositol oxygenase, putative, Os1bglu4-β-glucosidase-like protein without signal sequence and hsp20/α-crystalline family protein (**Figure 6** and **Supplementary Figures S4** and **S5**). *R. solani* treatment resulted in a larger number of genes (92) being differentially expressed. As many as 19 genes were >twofold up-regulated and many of the up-regulated genes were clearly related to defense and pathogen related stress. The highly up-regulated genes were: oxidoreductase, aldo/keto reductase family protein, OsWAK14-OsWAK receptor-like protein kinase, ankyrin repeat domain containing protein, matrix attachment region binding protein, peptide transporter PTR2, retrotransposon protein, sulfate transporter, putative dehydration-responsive element-binding protein, ethylene-responsive transcription factor and putative serine/threonine protein kinase. In SN13 + *R. solani* treatment, a large number of genes (90) were differentially regulated significantly, however, none of the genes were >1.5-fold up-regulated and only three genes were >1.5-fold down-regulated (**Supplementary Figure S5**). There were only 10 genes that up-regulated > onefold. Interestingly the stress responsive genes were significantly down-regulated. The major genes up-regulated were: phospholipase D, auxin response factor, galactosyltransferases, etc. The genes were categorized based on functional pathways involving primary metabolism, stress responses and hormone biosynthesis. Many genes in different categories show very contrasting expression profiles. The arrows in **Figure 6** show those genes in SN13 + *R. solani* which has been restored to control levels as compared to *R. solani* alone treatments.

**FIGURE 7:**
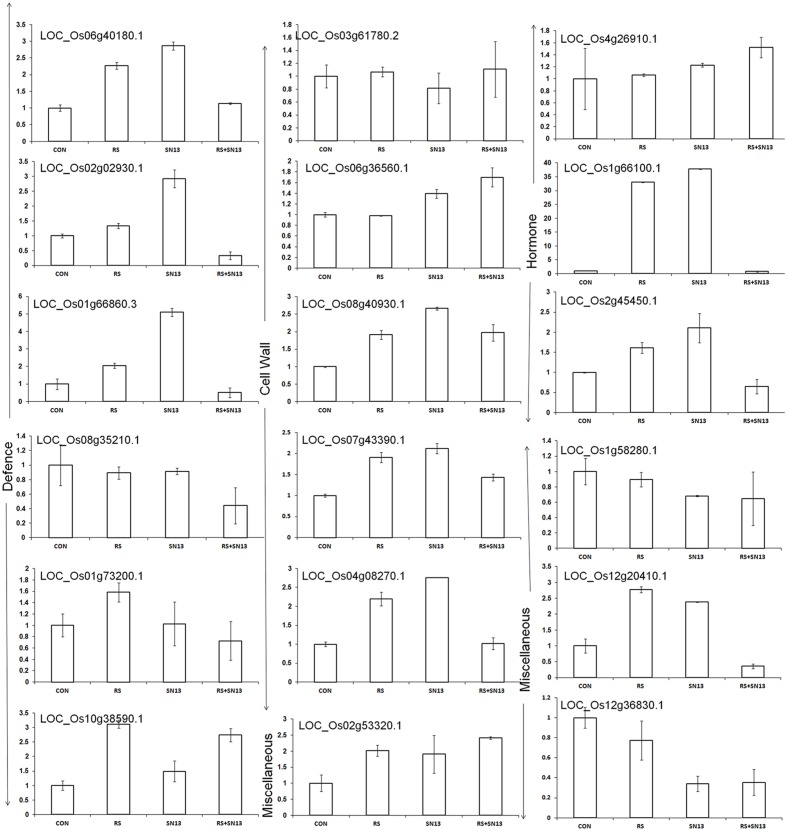
**RT-PCR analysis of randomly selected genes in rice leaves 45 dpi.** CON, control; SN13, biocontrol agent; *R. solani*, pathogen.

Validation of microarray results through RT-PCR analysis of randomly picked genes (marked as blue arrow in **Figure 6**) was performed using gene specific primers. The Analysis of genes related to cell wall disintegration, limit dextrinase (Os04g08270), 4-α-glucan transferase (Os07g43390), isoamylases (Os08g40930), and inositol oxygenase (Os06g36560) showed up-regulation in *R. solani* treatment as compared to control. Up-regulation of isoamylases and 4-α-glucan transferase in SN13 + *R. solani* treatment was also observed. In a further support to this Os03g61780, coding for glucan endo-1,3-β-glucosidase showed marginal overexpression, contrary to the microarray result (-1.2-fold down-regulation) in fungus treatment as compared to control. Universal stress protein domain (Os02g53320) was another gene up-regulated in SN13 as compared to control (**Figure 7**). The pathogenesis related Bet V gene (Os12g36830) was down-regulated in *R. solani* and SN13 treatment whereas it was further down-regulated in SN13+*R. solani* treatment. Down-regulation of Bet V gene was very well correlated in both the experiments.

Further, overexpression of serine threonin protein kinase (Os 01g66860) of MAPKinases family by fivefold and phospholipase D (Os06g40180) involved in signaling of plants’ multiple defense response showed up-regulation by threefold in SN13 as compared to control and twofold as compared to *R. solani*. Gene responsible for ROS balancing, Os08g35210 coding for ferric reductases of plasma membrane NADPH oxidase family (Nox) was down-regulated (0.5-fold) in SN13 + *R. solani* treatment (**Figure 7**). However, genes coding for peroxidase precursor (Os01g73200) and glutathione *S*-transferase (Os10g38590) were found to be up-regulated in *R. solani* alone treatment as compared to control and further down-regulation of these genes in SN13 + *R. solani* treatment was observed, which does not correlate with the microarray results.

Up-regulation of gibberellin 20-oxidase (Os01g66100) and a DREB homolog (Os02g45450), genes in SN13 (∼2.5-fold) and *R. solani* treatment (∼1.5-fold) has been found up-regulated in present study which shows further similar expression as compared to control in SN13 + *R. solani* treatment. The gene (Os04g26910) encoding an oxidoreductase showed concomitant increase in *R. solani*, SN13 and SN13 + *R. solani* treatment predicting increased auxin as a defense response.

Matrix attachment binding protein (Os12g20410) existing in a co-repressor/co-activator complex was up-regulated in fungus (3.2-fold) and bacterial treatment (2.1-fold) but was down-regulated in the SN13 + *R. solani* treatment. Role of stomatal closure was also observed by the down-regulation of subtilisin homolog (Os01g58280) in *R. solani* and SN13 and similar level of expression of this gene in SN13 + *R. solani* was observed as a stress reliever (**Figure 7**). Differential accumulation of metabolite observed was also correlated with the overexpression of terpene synthase (Os02g02930) in SN13 (threefold) well correlated with the accumulation of quinazoline due to beneficial interaction.

## Discussion

The severity of sheath blight disease in rice depends on many external and internal factors such as plant vigor, presence of beneficial microbes and nutritional status of the soil (Herman et al., 2008; Srivastava et al., 2012; Carvalhais et al., 2013). Present study elucidates the effect of a bioinoculant (SN13), a pathogen (*R. solani*) and their interaction (SN13 + *R. solani*) on the network of events active at a relatively later stage of infection in rice plants, unlike in other studies where immediate events are discussed. The chain of events involved participation of the plant’s machinery related to enhanced elicitation, ROS scavenging, hormonal cross talk, sugar signaling, and secondary metabolism. Correlations among different events have been discussed in this study.

*Bacillus amyloliquefaciens* (SN13) antagonism of *R. solani* under *in vitro* conditions mediated by mycolytic enzymes was increased in SN13 + *R. solani* which was probably triggered by presence of *R. solani* as reported earlier (Smitha and Singh, 2014). Effect of this interaction was evident under greenhouse conditions in terms of reduced fungal colonization or biocontrol (Frey-Klett et al., 2011) and presence of a constant source of elicitors.

SN13 imparted health and vigor to the plants with or without *R. solani* and enhanced carbon assimilation can be related well with the higher dry mass, chlorophyll content, and starch accumulation (**Table 1**; **Figure 3**). Induction of PG and PME after 15 days of infection in *R. solani* treatment probably resulted in cell wall loosening through degradation and desertification of pectins (Raiola et al., 2011; Bellincampi et al., 2014). Down-regulation of PME and up-regulation of PG and β-gluc activity (oligosaccharide release) in SN13 primed plants probably enhanced deliverance of elicitor responsible for the induced systemic response. However, this activity declines with time as supported by the down-regulated genes in microarray data and also attenuated enzyme activities after 45 dpi (**Figure 4A**).

**FIGURE 8:**
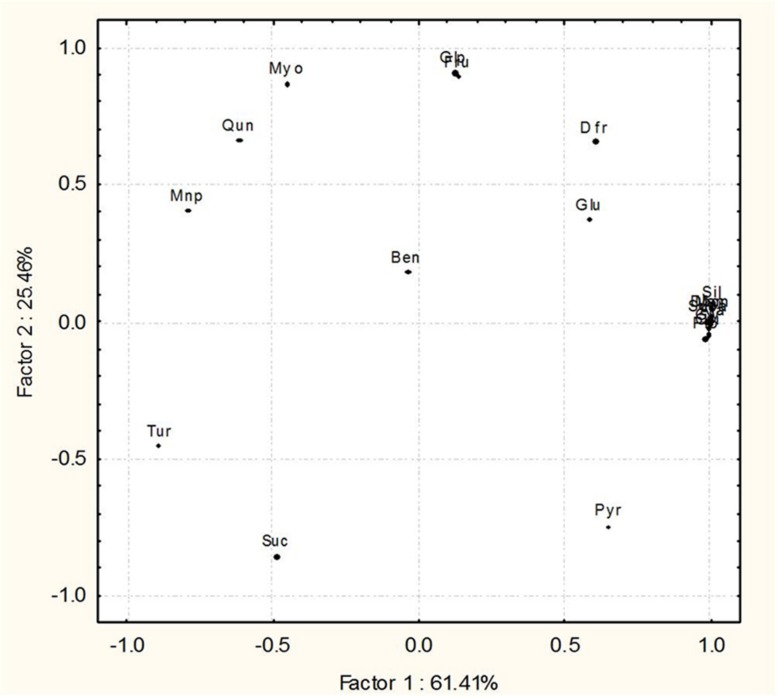
Principal component analysis (PCA) differentiating the components of GC–MS metabolic profiles of *O. sativa* leaves 45 dpi in the four treatments, control, pathogen infected (*R. solani*), *Bacillus amyloliquefaciens* treated (SN13) and pathogen + *B. amyloliquefaciens* (SN13 + *R. solani*).

A consistency in CWDE in *R. solani* infected plants, is congruous with the fact that pathogens first invade the cell wall through differential expression of limit dextrinase (Os04g08270), 4-α-glucan transferase (Os07g43390), isoamylases (Os08g40930), and inositol oxygenase (Os06g36560) which have role in cell wall expansion, starch and sucrose metabolism and oxidation of myoinositol to glucuronic acid (Trafford et al., 2013) was also correlated well with myoinositol content. Transcriptional and translational correlation between myoinositol concentration and inositol oxygenase (Os06g36560) exists in SN13 + *R. solani* and SN13 treatments probably maintains a high glucuronic acid concentration for elicitation of defense (Figures 5, 6 and 8; Supplementary Table S2; Vera et al., 2011). Higher myoinositol might result in hypersensitive response by inducing local cell death and suppressing effector triggered immunity through R gene (Os11g46070) and disease resistance protein interaction (Os06g06850; **Figure 6**) (Bruggeman et al., 2015). Furthermore proteases from SN13 may be inducing nitrogen limitation for fungal partner and induce immune response as reported for proteases from pathogenic sources during sheath blight disease (Suarez et al., 2005; Zheng et al., 2013). The observations clearly demonstrate role of SN13 in employing plants’ system to elicit defense that not only maintains the cell wall integrity but also resists pathogen invasion by degrading fungal cell wall polysaccharides, as evident earlier (Nakano et al., 2013).

A stable and balanced intracellular redox is maintained by the plant’s defense mechanism by differentially regulating various enzymes and/or metabolites (Hare and Cress, 1997). Modulation of proline levels by SN13 and *R. solani* in different treatments affirmed as an indicator of ROS status; lowered proline and ROS in SN13 and higher in SN13 + *R. solani* treatments shows competence of the bacteria to maintain ROS balance as per requirement of the plant (**Table 1**) (Hare and Cress, 1997). An induced level of ROS quenching enzymes such as CAT, APX and SOD in *R. solani* treatment an indicator of high intracellular redox is in concordance with the up-regulation of genes coding for peroxidase precursor (Os01g73200) and glutathione *S*-transferase (Os10g38590). Controlled intracellular redox in SN13 + *R. solani* is in accordance to the down-regulation of defense responsive genes and enzymes. Down-regulation of NADPH oxidase family such as ferric reductase (Os08g35210), a key producer of ROS in SN13 + *R. solani* (0.5-fold) emphasizes relatively unstressed conditions (**Figure 6**) as stated earlier (Nakano et al., 2013). These cell wall interactions and elicitation lead to kinase mediated signaling, as evident by overexpression of serine-threonin protein kinase (Os01g66860) of MAPKinases (fivefold) and phospholipase D (by threefold in SN13 as compared to control) that functions in response to ROS accumulation. The results are in accordance to prior report where enhanced level of these genes was associated with pathogen resistance (Sun et al., 2014). Differential accumulation of ROS quenchers like pyrazole, imidazole, and chlorothiophene in SN13 + *R. solani* as compared to *R. solani* treatment also insist SN13 mediated ROS balancing. The sustained plant defense may thus be attributed to the other unconventional mechanisms.

Levels of metabolizable sugars (glucose/fructose) in SN13 + *R. solani* may be responsible for inducing auxin, however, their enhanced production in *R. solani* and SN13 alone may also be associated with increased ROS production and involvement of stress mediated signaling as also evident from microarray and RT-PCR analysis of auxin response factor 9 (Os4g36054; **Figure 7**) (Hamzehzarghani et al., 2005; Li et al., 2011; Sairanen et al., 2012; Nafisi et al., 2015; Naseem et al., 2015). Decreased SA accumulation in *R. solani* treatment corresponds well with prior reports of rice–necrotroph interaction (Bari and Jones, 2009). Increased SA level in SN13 treatments reinforces the role of SN13 in priming the defense response active both in presence and absence of pathogen. Higher level of ABA and GA in SN13 + *R. solani* treatment propose ABA and GA mediated defense regulation in plants as evident in microarray data and earlier reports against *Pythium irregulare* and *Magnaporthe grisea* (Figures 5 and 6) (Ton and Mauch-Mani, 2004; Schmidt et al., 2008; Parker et al., 2009; Zheng et al., 2013; Nafisi et al., 2015). The delayed aerenchyma formation (probably due to low ABA and GA) and less starch content in parenchyma cells (anatomical results) emphasize SN13 mediated delayed apoptosis of parenchyma cells even under high oxidative state for improved stress tolerance through the overexpression of universal stress protein domain (Os02g53320, coding for ENOD18) having role in adjusting hypoxic conditions through differential hormone signaling and accumulation of ROS quenchers like pyrazole, imidazole, and chlorothiophene (Sauter et al., 2002).This may be argued as a probable mechanism of biocontrol by PGPR in plants and opens a new avenue of further verification.

The interplay of hormones and metabolites employed by SN13 primed plants when challenged with a pathogen lead to a stark difference in the accumulation of sugars, their alcohols and pyranoses as compared to earlier report of defense elicitation (Zhang et al., 2015). These differences were observed as widely separated metabolites on the PCA plot which may be used as predictive marker metabolites to understand the effects of PGPR mediated amelioration of biotic stresses (**Figure 8**; **Supplementary Figure S6**). The high levels of glycerol, mannitol, and arabitol cause the quenching of ROS generated by plant, thereby acting in plant defense and also act as nutrient for *R. solani* as supported by Zhang et al. (2015) (**Figure 8**; **Supplementary Figure S3** and Supplementary Table S2). Enhanced amounts of pyranoses in SN13 + *R. solani* provided a pool of sugars which are easily inter-convertible to usable sugar when required but not available for ROS generation (**Figure 8**). Pyranoses are also known to be the cyclic sugars that will polymerize for the formation of storage molecules like starch. Therefore, it may be hypothesized that one of the mode of action of SN13 primed plants to combat an infection is by depriving the pathogens for readily usable forms of sugars (**Figure 8**). A reduction in sucrose/hexose (glucose + fructose) ratio in *R. solani* (0.56) and SN13 (0.75) and further enhancement in SN13 + *R. solani* (0.79) treatment is in accordance to the report that cell wall invertases play crucial role for determining the pathways, differently triggered by pathogen and PGPR through fine regulation of sucrose/hexose ratio in an ABA dependent manner (Figures 5 and 8) (Bolouri-Moghaddam et al., 2010; Li et al., 2011; Tauzin and Giardina, 2014). Up-regulation of α-amylases (Os08g40930) and 4-α-glucanotransferase (Os07g43390) in SN13 + *R. solani* treatment correlates with decreasing sucrose (GC MS results) and starch content (anatomical results) showing importance of SN13 in modulating starch and sucrose metabolism (Figures 3 and 8; Supplementary Table S2).

Differences in yet another sugar, turanose, a non-metabolizable sucrose analog is of interest since its active involvement during plant pathogen interaction has not been reported earlier to the best of our knowledge. Reduced accumulation of turanose in *R. solani* (∼90%) intends pathogen mediated inhibition of defense signaling pathway by catabolizing turanose to produce osmolytes, glutamate and the dipeptide *N*-acetylglutaminyl glutamine amide (Mchunu et al., 2013) (**Figure 8**; **Supplementary Figure S3** and Supplementary Table S2). The negative correlation observed between turanose and proline in *R. solani* treatment may be due to its catabolism to glutamate which is precursor for proline. The observation opens a new area of investigation in determining role of turanose in plant–microbe interactions.

Exclusive production of propionic and succinic acid of TCA cycle during *R. solani* interaction may indicate a compensatory response to the requirement for a high metabolic flux during plant pathogen interaction to cope up the increasing energy consumption (Alteri et al., 2015). Accumulation of tryptophan dependent quinoline, an alkaloid of phenylpropanoid pathway emphasizes the switch between primary to secondary metabolism during pathogen interaction (Iriti and Faoro, 2009). Overexpression of terpene synthase (Os02g02930; involved in terpene biosynthesis) is correlated well with various defense mechanisms and improved biological control (Tholl et al., 2011). The threefold up-regulation in SN13 is well correlated with the accumulation of antibacterial like benzene dicarboxylic acid and quinazoline resulting in sustained defense response (**Supplementary Figure S3**).

Up-regulation of genes related to biosynthetic processes, catalytic activity and transferase activity during *R. solani* infection are indication of plant’s agitated condition. Differentially expressed genes in SN13 are characteristic of plant–bacterial interaction and down-regulation of defense related genes emphasize plant’s rather unstressed environment. The GO terms of SN13 + *R. solani* are comparable to plants treated only with SN13which is also evident in the PCA plot with metabolites (**Supplementary Figure S6**).

From the above discussion it is evident that SN13 primed and unprimed plants responded differently upon *R. solani* challenge. The bioinoculant SN13 induced a network of events which resulted in long term priming of the plants against the infection. Direct confrontation of the pathogen enhanced elicitation of immune response was through the maintenance of differential physiological and metabolic status. ROS modulation, either through accumulation of quenchers (mannitol, arabitol, and phospholipases) or through generation of ROS inhibitors was observed. SN13 primed plants exhibited rare sugars (pyranoses and turanose), which eventually compromised the fungal growth and elicited defense response using MAPK signaling and fortification of metabolites like terpenes and quinazoline. Auxin responsive hormonal cross talk in SN13 induced resistance enhancing plant immunity in ISR mediated SAR in SA, ABA, and benzene dicarboxylic acid dependent manner where expression of JA (Os03g55800) and ethylene responsive transcription factor (Os03g09170) remained unaltered (**Figure 6**).

## Conclusion

The present study shows a sustained tolerance toward *R. solani* infection by SN13 primed plants, that may be attributed to unconventional mechanisms mediated through (a) general maintenance of plant health and vigor, (b) involvement of bacterial mycolytic enzymes, (c) cell wall modification and sustained maintenance of elicitors, (d) a delicate balance of ROS and ROS scavengers that helped in both resisting the pathogen infection and protecting its own cells and, (e) production of metabolites especially non-metabolizable sugars and secondary metabolites.

## Author Contributions

SuS, VB, SoS, PS, did all the experiments. PKT and MA, assisted in analysis of Microarray experiment. SuS and PS, wrote the MS. PSC, PKT, and CSN reviewed the manuscript.

## Conflict of Interest Statement

The authors declare that the research was conducted in the absence of any commercial or financial relationships that could be construed as a potential conflict of interest.

## Supplementary Material

FIGURE S1 **Light micrograph showing colonization of rice leaves by *R. solani* during infection in absence (C,E,G) and presence of a biocontrol/PGPR SN13 (D,F,H) on stem surface in different treatments; control (A, CON), *Bacillus amyloliquefaciens* NBRISN13 (B, SN13), pathogen treatment (*R. solani*) and combination of both (SN13 + *R. solani*) after 15 dpi.** Magnification 10×; **A–D**: 4×; **E–F:** 10×; **G–H**: 40×.

FIGURE S2 **HPLC chromatogram of standards, 1 = gibberellic acid, 2 = indole acetic acid, 3 = abscisic acid, 4 = salicylic acid and of Control; *R. solani*; SN13 and SN13 + *R. solani* treatments of rice leaves after 15 days of infection**.

FIGURE S3 **Comparative ion electropherograms of metabolites in methanolic leaf extracts of rice through GC–MS analysis in absence (CON) and presence of sheath blight fungi (*R. solani*) and PGPR *B. amyloliquefaciens* (SN13) and combination of both (SN13 + *R. solani*)**.

FIGURE S4 **Expression profiling and hierarchical clustering of differentially expressed genes obtained through microarray data when comparisons were made between (A) Control_Fungus (CON_*R. solani*), (B) Control_Bacteria (CON_SN13) and, (C) Control_Bacteria + Fungus (CON_SN + *R. solani*)**.

FIGURE S5 **Functional classification of the up-regulated (a) and down-regulated (b) *Oryza sativa* genes in terms of their Gene ontology (GO terms), relative to their representation in the genome of rice plant, involved in a plant, PGPR, and pathogen interaction**.

FIGURE S6 **Principal component analysis (PCA) of rice treatments differentiating control, pathogen infected (*R. solani*), *Bacillus amyloliquefaciens* treated (SN13), and pathogen + *B. amyloliquefaciens* (SN13 + *R. solani*) treatments based on GC–MS metabolic profiles of leaves**.
