# Supplementary material for: Unraveling Aspects of Bacillus amyloliquefaciens Mediated Enhanced Production of Rice under Biotic Stress of Rhizoctonia solani
Source: Front Plant Sci. 2016 May 6;7:587. doi: 10.3389/fpls.2016.00587 (PMC4858605; doi:10.3389/fpls.2016.00587)
Supplement: Supplementary file 1 [file Data_Sheet_1.DOC]

Unraveling aspects of *Bacillus amyloliquefaciens* mediated enhanced production of rice under biotic stress of *Rhizoctonia solani*

Suchi Srivastava, Vidisha Bist, Sonal Srivastava, Poonam C. Singh, Prabodh Kumar Trivedi, Mehar Hasan Asif, Puneet Singh Chauhan, Chandra Shekhar Nautiyal*


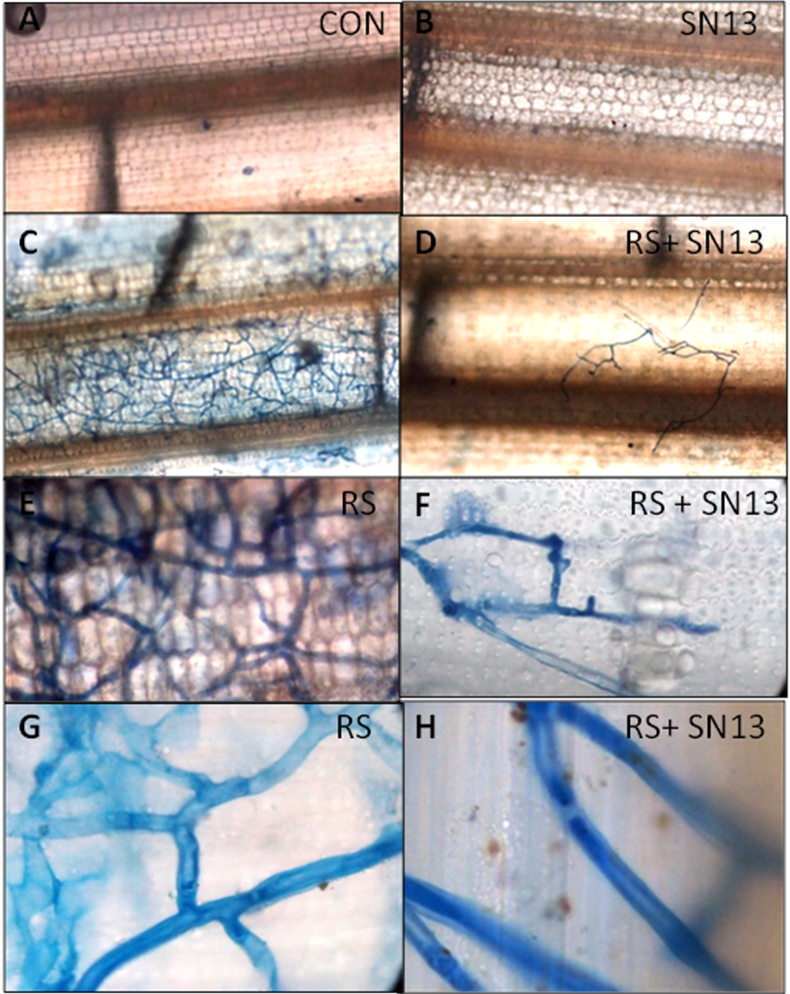


FIGURE S1A| Light micrograph showing colonization of rice leaves by *R. solani* during infection in absence (C,E,G) and presence of a biocontrol/PGPR SN13 (D,F,H) on stem surface in different treatments; control (CON), *Bacillus amyloliquefaciens* NBRISN13 (SN13), pathogen treatment (RS) and combination of both (SN13+RS) after 15 dpi. Magnification 10X; A-D:4X ; E-F:10X ; G-H:40X.

FIGURE S2 | HPLC chromatogram of standards, 1 = gibberellic acid, 2 = indole acetic acid, 3 = abscisic acid, 4 = salicylic acid and of Control; RS; SN13 and SN13+RS treatments of rice leaves after 15 days of infection.

FIGURE S3 | Comparative ion electropherograms of metabolites in methanolic leaf extracts of rice through GC-MS analysis in absence (CON) and presence of sheath blight fungi (RS) and PGPR *B. amyloliquefaciens* (SN13) and combination of both (SN13+RS).

A

B

C


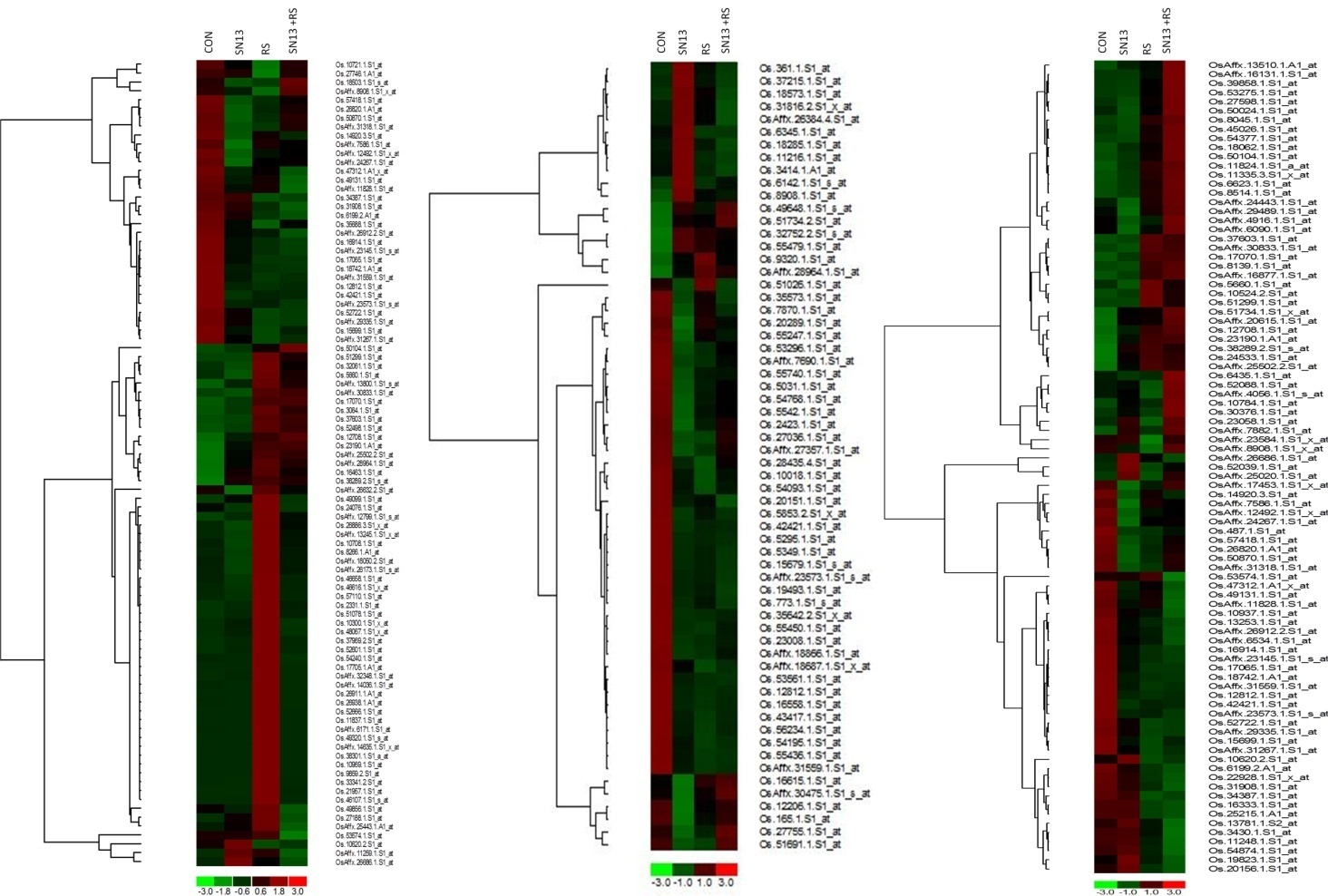


FIGURE S4 | Expression profiling and hierachial clustering of differentially expressed genes obtained through microarray data when comparisons were made between control_fungus (CON_RS) (A) control_bacteria (CON_SN13), (B) and control_bacteria+fungus (CON_SN+RS) (C).

FIGURE S5 | Functional classification of the up regulated (a) and down regulated (b) *Oryza sativa* genes in terms of their Geneontology (GO terms), relative to their representation in the genome of rice plant, involved in a plant, PGPR and pathogen interaction.

FIGURE S6 | Principal component analysis (PCA) of rice treatments differentiating control, pathogen infected (RS), *Bacillus amyloliquifaciens* treated (SN13) and pathogen+ *B. amyloliquifaciens* (SN13+RS) treatments based on GC-MS metabolic profiles of leaves.

| Table S1 List of primers of randomly selected genes for real time validation     | Category | Gene name | LOC No. | Primer sequence | | --- | --- | --- | --- | | Defense | Phospholipase D | Os06g40180 | For: CACCTG AACGTCAATG GGCAG  Rev: TCTCCGGACACACTCCAGGC | | Hormone | Terpene synthase | Os02g02930 | For: ACCGGGAGTGCTTCTCCGCTAG  Rev: GCCGTCGTAGCCATACATGACG | | Serine threonine protein kinase | Os01g66860 | For: TTATCAAGCTGTATTCCGAGGA  Rev:CTTTAGGTCGCAGTGGATGATGG | | Ferric reductase | Os08g35210 | For: GCTGCACAACTATCTCACAAGT  Rev: AGGCCTTGCAAAATGTGTCCTG | | Peroxidase precursor | Os01g73200 | For: CGACCCCACCATGGACAAGTG  Rev: TGGCGGTTTTGCAGGTCGACG | | Glutathione S-transferase | Os10g38590 | For: GACGAGCTCATGAAGCAGACGC  Rev: CAGCACGACGTCGACGTAGCCG | | Auxin induced family protein | Os04g26910 | For: TTGGAGCACTATCTGTGAAGCT  Rev: CCAAGTAGTGTTGGCCATCGC | | Cell wall | Gibberelin 20-oxidase | Os01g66100 | For: TCGAACGGGAGGTATAAGAGCT  Rev: CTGCGGCGTGGCGGCGCTCG | | DREB homologue | Os02g45450 | For: ATGCAGGGCTATCTCGACATG  Rev: CAGTAGCTCCATAGCTGGACC | | β-glucosidase | Os03g61780 | For: AACTCGATGTACGGCTCCGACA  Rev: ACACTTTCTCCTTGACATATGC | | Miscellaneous | Inositol oxygenase | Os06g36560 | For: GAGAACCCTGACTACCTGAAC  Rev: CTTGTTCTCCTTGGCAACCAGG | | Isoamylase | Os08g40930 | For: CATGGCCATCAGCCTGGGAAA  Rev: CAACCGCCGGCAAATGGCTGG | | α-glucan transferase | Os07g43390 | For: TGGCGGTGGTTCTGACAATCC  Rev: AGTCTGCTTCTCTTCCTCTGG | | Limit Dextrinase | Os04g08270 | For: GGTCCCTCGATGGTACCAGGAG  Rev: ACAGACATTGAAGATCGTCACG | | Universal stress protein | Os02g53320.1 | For: GGCAGTAGACGATCTCAAGGTG  Rev: CACCACAGTAACCGGGCAGGTT | | Subtilisin homologue | Os01g58280 | For: GGTCTCATGCTGCTCTCAAATC  Rev: AGGATTTATGTTCCCTCCTCC | | Matrix attachment binding protein | Os12g20410 | For: TGATGAAACTTGCTACGGGTA  Rev: CTTCCCTTCATACTTATGTGC | | PR10 family protein | Os12g36830 | For: TGGAAGGTCTTCTCCGACGCGC  Rev: ATCATCCACAGCAGGGTTGAGC | | Auxin responsive | Os04g36054.1 | For:CTCCTACGAGGAGGCCAAGCTGC  Rev: AGGCATGATTGTCCAGGTCGAC | |  | OsActin | For: GAGTATGATGAGTCGGGTCCAG  Rev: ACACCAACAATCCCAAACAGAG | |  |  |
| --- | --- | --- | --- | --- | --- | --- | --- | --- | --- | --- | --- | --- | --- | --- | --- | --- | --- | --- | --- | --- | --- | --- | --- | --- | --- | --- | --- | --- | --- | --- | --- | --- | --- | --- | --- | --- | --- | --- | --- | --- | --- | --- | --- | --- | --- | --- | --- | --- | --- | --- | --- | --- | --- | --- | --- | --- | --- | --- | --- | --- | --- | --- | --- | --- | --- | --- | --- | --- | --- | --- |

Table S2 Metabolic profiling of *O. sativa* leaves in absence (CON) and presence of *B. amyloliquefaciens* (SN13), *R. solani* (RS) and combination of PGPR+ pathogen (RS+SN13) after 45 dpi

| RT | Name | Nature of the compound | Peak Area % | | | | Distinctive functions observed in relation to pathogen infection | Reference(s) |
| --- | --- | --- | --- | --- | --- | --- | --- | --- |
|  |  |  | CON | RS | SN | RS+SN |  |  |
| 6.63 | Propanoic acid | Carboxylic acid | 0.14±0.02 | 0.46±0.01 | 0.07±0.02 | 0.12±0.00 | Act as growth inhibitor | Armstrong and  Armstrong, 1999 |
| 10.95 | Silanamine | Sesqueterpenes | 0.03±0.00 | 0.20±0.00 | 0.02±0.00 | 0.03±0.00 | Higher in potato after phyotphthora infection | Cheng et al., 2007 |
| 11.58 | 1H-Pyrazole | Heterocyclic organic compound | 5.13±0.09 | 5.54±0.02 | 4.20±0.10 | 3.05±0.04 | Act as an inducer for SAR | Yasuda et al., 2003 |
| 12.4 | 1H-Imidazole | Heterocyclic organic compound | 0.00±0.00 | 16.38±0.07 | 0.00±0.00 | 0.01±0.00 | Inhibitor of ROS generation in a NADPH oxidase dependent; imidazole alkaloids from histidine during defence response | Iriti and Faoro, 2009 |
| 13.85 | Succinic acid | Dicarboxylic acid | 0.00±0.00 | 3.79±0.72 | 0.00±0.00 | 0.00±0.00 | Higher content in susceptible plant during pathogen interaction | Leiss et al., 2009 |
| 18.39 | Glycerol | Sugar alcohol | 0.02±0.00 | 0.13±0.01 | 0.02±0.00 | 0.01±0.00 | Application of glycerol as a foliar spray activates the defence response and enhances disease resistance | Zhang, 2015 |
| 21.53 | Arabitol | Sugar polyols | 0.07±0.00 | 0.455±0.02 | 0.07±0.00 | 0.06±0.00 | Diagnostic marker for invasion, defence for quenching the reactive oxygen species | Link et al., 2005 |
| 22.24 | Mannitol | Sugar polyols | 0.00±0.00 | 0.385±0.00 | 0.00±0.00 | 0.06±0.00 | Mannitol, at least, functions in the translocation of carbohydrates from plant roots to their fungal symbionts, defence for quenching the reactive oxygen species | Link et al., 2005 |
| 22.67 | 2,5-di-butyl-3-Chlorothiophene | Heterocyclic aromatic compound | 0.00±0.00 | 0.97±0.00 | 0.015±0.015 | 0.01±0.01 | Superoxide inhibitory and free radical scavenger activity | Mandawad et al., 2013 |
| 23.22 | Fructopyranose | Carbohydrate | 0.34±0.00 | 3.33±0.04 | 1.625±0.19 | 5.91±0.77 | Act as an elicitor for plant defence | Hamzehzarghani et al., 2005 |
| 25.16 | d-fructose | Ketonic monosaccharide | 5.08±0.09 | 7.85±0.04 | 7.34±0.17 | 6.82±0.09 | Involved in stress mediated signalling | Cho and Yoo, 2011; Li et al., 2011 |
| 25.69 | d-glucose | Sugar | 13.58±1.2 | 23.99±0.02 | 23.15±3.43 | 16.84±0.2 | High glucose is associated with toxicity and pathogenesis through increased production of ROS by glucose auto oxidation and glucose metabolism | Russell et al., 2002; Barros et al., 2004, Hamzehzarghani et al., 2005 |
| 26.77 | Mannopyranose | Sugar monomer | 2.26±0.00 | 0.56±0.08 | 5.20±0.38 | 3.40±0.35 | Act as an elicitor for plant defence | Hamzehzarghani et al., 2005 |
| 27.42 | β-d glucopyranose | Carbohydrate | 0.16±0.02 | 3.90±0.00 | 1.95±0.00 | 7.2±0.66 | Act as an elicitor for plant defence | Hamzehzarghani et al., 2005 |
| 28.32 | Myoinositol | Carbohydrate | 0.18±0.00 | 0.24±0.03 | 0.62±0.14 | 1.27±0.05 | Myoinostol biosynthesis correlates well with programmed cell death | Hamzehzarghani et al., 2005; Eckardt, 2010 |
| 36.35 | Quinoline | Heterocyclic aromatic organic compound | 0.01±0.00 | 0.1±0.01 | 0.01±0.00 | 0.01±0.00 | An alkaloid plant secondary metabolite gets synthesized during plant defence from tryptophan | Iriti and Faoro, 2009 |
| 37.71 | Sucrose | Disaccharide | 33.29±0.77 | 17.94±0.07 | 22.90±0.38 | 18.60±0.35 | Due to the increased activity of invertase during infection sucrose gets hydrolysed in glucose and fructose and serve as nutrient for the invader. Signalling molecule for plant innate immunity | Parker et al., 2009 |
| 38.45 | Turanose | Non metabolizable sugar (analogue of sucrose) | 25.81±0.35 | 2.28±0.36 | 18.60±0.74 | 15.38±0.71 | Non metabolizable Sucrose analogue known to elicit stress responses in plants | Roitsch et al., 2003 |
| 42.67 | 1,2-Benzene dicarboxylic acid | Aromatic dicarboxylic acid | 0.04±0.00 | 0.05±0.00 | 0.06±0.02 | 0.05±0.02 | Plant activator to enhance salicylic acid production | Rahmawati et al., 2014 |
| 44.64 | Quinozoline | Heterocyclic aromatic organic compound | 0.01±0.00 | 0.01±0.00 | 0.03±0.01 | 0.04±0.01 | An alkaloid synthesized from the precursor anthranilic acid during plant defence response | Iriti and Faoro, 2009 |

- ****Armstrong, J., and Armstrong, W.****(**1999**).**Phragmites**die-back: toxic effects of propionic, butyric and caproic acids in relation to pH.***New Phytol.* 142, 201**–217.
- Barros, M.H., Bandy, B., Tahara, E.B., and Kowaltowksi, A.J. (2004). Higher respiratory activity decreases mitochondrial reactive oxygen release and increases life span in *Saccharomyces cerevisiae*. *J. Biol. Chem.* 279, 49883–49888.
- ****Cheng, A.X., Lou, Y.G., Mao, Y.B., Lu, S., Wang, L.J., and Chen, X.Y.****(2007). Plant terpenoids: Biosynthesis and ecological functions (Invited Review) **J. Integr. Plant Biol. **49****, 179–186.
- Cho, Y.H., and Yoo, S.D. **(**2011). Signaling role of fructose mediated by FINS1/FBP in *Arabidopsis thaliana*. *PLoS Genet,* 7, e1001263.
- Eckardt. **(2010). Myo**-Inositol biosynthesis genes in**Arabidopsis**: Differential patterns of gene expression and role in cell death. ***Plant Cell.* 22, 537.**
- Hamzehzarghani, H., Kushalappaa, A.C., Dionb, Y., Riouxc, S., Comeaud, A., Yaylayane, V., Marshalle, W.D., and Mather, D.E. (2005). Metabolic profiling and factor analysis to discriminate quantitative resistance in wheat cultivars against *Fusarium* head blight. *Physiol. Mol. Plant Pathol.* 66, 119–133.
- Iriti, M., and Faoro, F. (2009). Chemical diversity and defence metabolism: How Plants Cope with pathogens and ozone pollution. *Int. J. Mol. Sci.* 10, 3371–3399.

# Leiss, K.A., Maltese, F., Choi, Y.H., Verpoorte, R., and Klinkhamer, P.G.L. (2009). Identification of chlorogenic acid as a resistance factor for Thrips in *Chrysanthemum*. *Plant Physiol.* 150, 1567–1575.

- Li, P., Wind, J.J., Shi, X., Zhang, H., Hanson, J., Smeekens, S.C., and Teng, S. (2011). Fructose sensitivity is suppressed in *Arabidopsis* by the transcription factor NAC089 lacking the membrane-bound domain. *Proc. Natl. Acad. Sci.* 108, 3436-3441.
- Link, T., Lohaus, G., Heiser, I., Mendgen, K., Hahn, M., and Voegele, R. T. (2005). Characterisation of a novel NADP+dependent D-arabitol dehydrogenase from the plant pathogen *Uromyces fabae. Biochem. J.* 389, 289-295.

# Mandawad, G.G., Dawane, B.S., Beedkar, S.D., Khobragade, C.N., and Yemul, O.S. (2013). Trisubstituted thiophene analogues of 1-thiazolyl-2-pyrazoline, super oxidase inhibitors and free radical scavengers. *Bioorg. Med. Chem.* 21, 365-72.

- Rahmawati, S.L., Esyanti, R.R., and Gunaeni, N. (2014). The role of leaf extracts as Plant-activator to enhance Salicylic acid production on Tomato plant (*Lycopersicon esculentum* Mill.) infected by CMV (*Cucumber Mosaic Virus*). *Int. J. Chem. Env. & Biol. Sci.* 2, 94-97.
- Roitsch, T., Balibrea, M.E., Hofmann, M., Proels, R., and Sinha, A.K. (2003). Extracellular invertase: key metabolic enzyme and PR protein. *J. Exp. Bot.* 54, 513-524.
- Russell, J., Golovoy, D., Vincent, A.M., Mahendru, P., Olzmann, J.A., Mentzer, A., and Feldman, E.L. (2002). High glucose-induced oxidative stress and mitochondrial dysfunction in neurons. *FASEB J.* 16, 1738-48.

# Yasuda, M., Nishioka, M., Nakashita, H., Yamaguchi, I., and Yoshida, S. (2003). Pyrazolecarboxylic acid derivative induces systemic acquired resistance in tobacco. *Biosci. Biotechnol. Biochem.* 67, 2614-20.

- Zhang, H., Cui, F., Wu, Y., Lou, L., Liu, L., Tian, M., Ning, Y., Shu, K., Tang, S., and Xie, Q. (2015). The RING finger ubiquitin E3 ligase SDIR1 targets SDIR1-INTERACTING PROTEIN1 for degradation to modulate the salt stress response and ABA signaling in *Arabidopsis*. *Plant Cell,* 27, 214-27.
